# Interfacial amplifier: High-throughput visual protein detection via droplet dynamics

**DOI:** 10.1126/sciadv.aef6559

**Published:** 2026-08-12

**Authors:** Tianxin Ge, Xuexue He, Shouzi Zhang, Haofeng Chen, Wenxu Hu, Zhaofan Luo, Liqiu Wang, Zong Dai, Xing Han

**Affiliations:** ^1^ Guangdong Provincial Key Laboratory of Sensing Technology and Biomedical Instrument, School of Biomedical Engineering, Shenzhen Campus of Sun Yat-sen University, Sun Yat-sen University, No. 66, Gongchang Road, Guangming District, Shenzhen, Guangdong 518107, P. R. China.; ^2^ The Seventh Affiliated Hospital, Sun Yat-sen University, Shenzhen, Guangdong 518107, P. R. China.; ^3^ Department of Mechanical Engineering, The Hong Kong Polytechnic University, Hong Kong 999077, Hong Kong, P. R. China.

## Abstract

Rapid, visual protein detection at ultralow cost remains a critical unmet need for scalable point-of-care testing. We introduce a high-throughput platform leveraging interfacial droplet dynamics on a slippery liquid-infused porous surface (SLIPS) to directly translate protein-specific adsorption into macroscopic behavior. Protein concentration and identity modulate the critical sliding angle of droplets via interfacial film formation, enabling pretreatment-free, instrument-free identification of clinically relevant targets like proteinuria within minutes. This approach achieves parallel analysis of 170 samples on a single A4-sized substrate (5 minutes, ∼$0.008 per sample), requiring only visual readout. By amplifying nanoscale interfacial interactions into intuitive visual outputs, our SLIPS-based technology establishes a scalable, user-friendly paradigm for decentralized diagnostics and continuous health monitoring across diverse settings.

## INTRODUCTION

Point-of-care testing (POCT) plays a pivotal role in modern healthcare by enabling rapid, portable, and cost-effective diagnostics (*1*–*3*). Yet, conventional POCT platforms face limitations in scalability and practicality for large-scale disease screening or chronic condition monitoring (*4*). Current colorimetric and fluorescence-based methods often rely on chemical reagents, which increase costs, complicate usability, and hinder long-term stability. These challenges underscore the urgent need for innovative, reagent-free, naked-eye detection technologies that balance affordability with precision. Interfacial interaction–driven systems have emerged as promising alternatives, leveraging liquid dynamics for visual readouts (*5*). Existing approaches include paper-based assays (*6*, *7*), droplet-motion detection (*8*, *9*), and liquid-gate-based detection (*10*, *11*). However, paper-based assays require pre-embedded reagents, limiting shelf life and adaptability. Droplet-motion methods correlate molecular properties (e.g., DNA length) with droplet movement on lubricated surfaces (*9*) but necessitate complex pretreatment steps, complicating home-use applications. Liquid-gate systems (*12*) translate interfacial effects into pressure signals, yet their scope remains confined to ion detection (*10*). Collectively, these gaps highlight the demand for a versatile, user-friendly platform capable of detecting biomolecules directly in untreated samples. Proteins in body fluids serve as important biomarkers for diseases such as kidney dysfunction (*13*) and dry eye syndrome (*14*). Traditional protein detection methods, however, struggle with balancing sensitivity, dynamic range, and operational simplicity. Bionics has brought many inspirations to various applications (*15*–*18*). Inspired by *Nepenthes alata* (*19*), a carnivorous plant with a naturally slippery surface, researchers have developed biomimetic lubricant-infused surfaces (*20*). These surfaces exhibit exceptional antibiofouling properties, minimizing residual biofluid adhesion even after prolonged exposure (*21*). While prior studies focused on short-term biofluid repellency, we hypothesized that the delayed protein adsorption on such surfaces could be harnessed for sensitive, quantitative detection.

In this study, we explore the dynamic behavior of protein droplets on slippery organogel surfaces following a resting period, aiming to correlate protein characteristics with macroscopic droplet motion. Building on prior research highlighting the exceptional antibiofouling properties of organogel substrates, we extend these findings by investigating time-dependent protein adsorption. Our experiments reveal that protein droplets exhibit an increased critical sliding angle (CSA) after resting, a phenomenon attributed to protein accumulation at both the water-oil interface and the solid surface. Notably, ionic strength and pH were found to exert negligible influence on CSA, underscoring the dominance of direct protein-surface interactions in governing droplet behavior. Leveraging these insights, we developed a diagnostic approach that translates microscale protein adsorption into measurable macroscopic CSA shifts. This method enables rapid, high-throughput, low-cost, pretreatment-free detection of proteinuria by visually tracking CSA changes, eliminating the need for complex instrumentation or signal amplification. We further validated the clinical feasibility of this strategy, demonstrating its potential as a cost-effective tool for point-of-care kidney disease screening and longitudinal monitoring. By bridging microscopic interfacial phenomena with user-friendly macroscopic readouts, this work advances accessible healthcare diagnostics through a simple, naked-eye-observable platform.

## RESULTS

### CSA of protein droplets

**Fig. 1.**
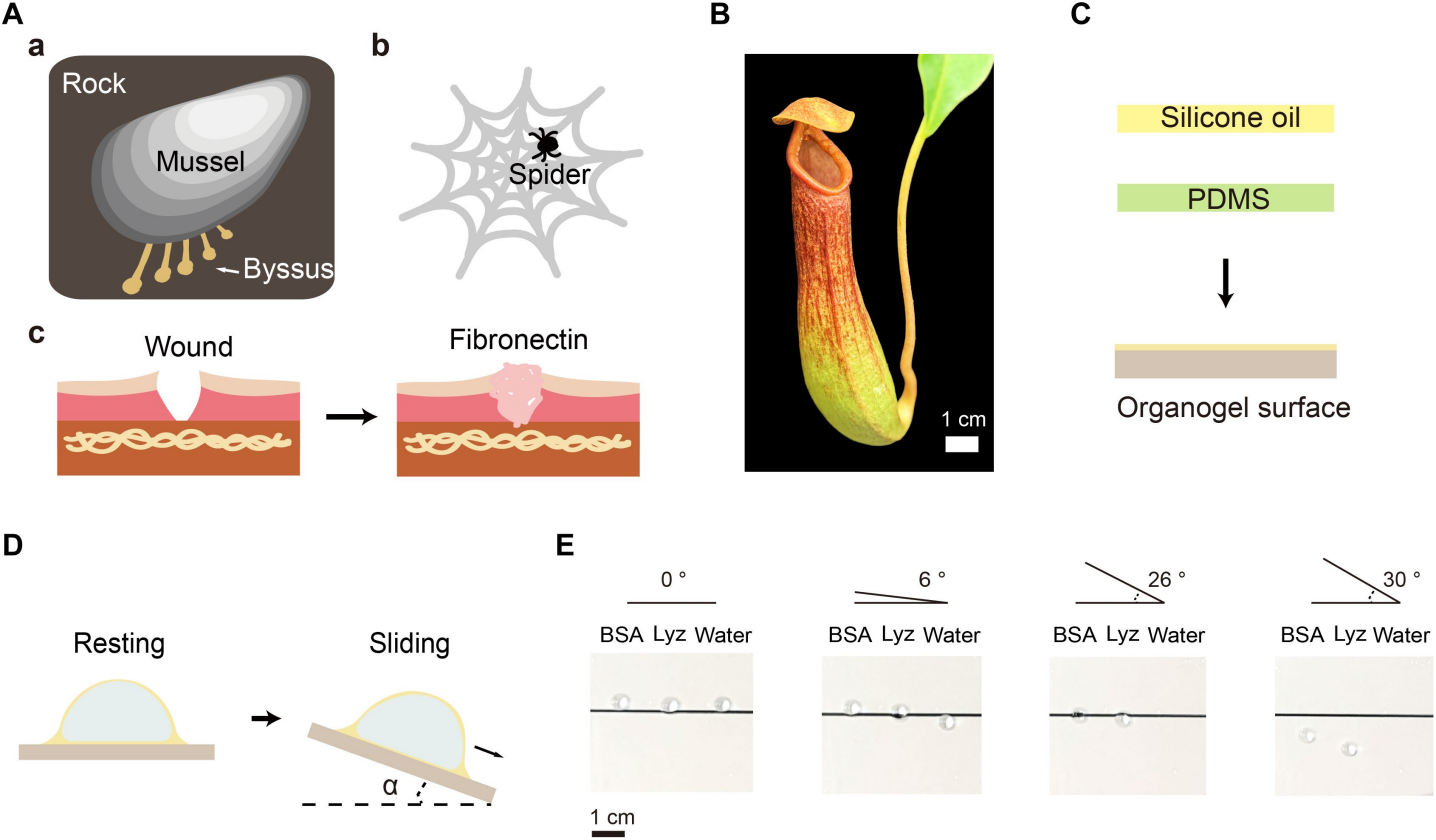
CSA as a macroscopic readout of protein adsorption on slippery organogel surfaces. (**A**) Protein adhesion mechanisms in nature: (a) mussels adhering to rocks via byssus proteins, (b) spiders spinning capture webs using silk proteins, and (c) fibronectin-mediated wound healing through cellular adhesion. (**B**) Optical image of the *Nepenthes pitcher* plant, the biological template for the slippery surface design. (**C**) Schematic of the organogel surface synthesis, highlighting its lubricant-infused porous structure. (**D**) Definition of the CSA (α), the tilt threshold at which droplets begin to slide. (**E**) CSA measurements of droplets (volume: 4 μl) after 20-min rest: Lyz (1 g liter^−1^, 26°), BSA (1 g liter^−1^, 30°), and pure water (6°), demonstrating protein-specific adhesion effects. (A) was created in BioRender. X. Han (2026) https://BioRender.com/ag7m6r9.

Protein adhesion to surfaces plays a pivotal role in diverse biological processes, enabling essential functions such as structural anchoring, material synthesis, and tissue repair (Fig. 1A). For instance, marine mussels secure themselves to rocky substrates using adhesive byssus proteins to resist turbulent waves (*22*), spiders spin robust capture webs through precisely tuned silk protein secretions (*23*), and fibronectin mediates cellular adhesion to accelerate wound healing (*24*). Building on these natural paradigms, we studied protein adhesion dynamics on bioinspired slippery liquid-infused porous surfaces (SLIPSs) (Fig. 1, B and C, fig. S1, and Methods and Materials), modeled after the *Nepenthes pitcher* plant’s lubrication strategy (*20*, *25*, *26*). In this system, a stabilized lubricant layer, anchored by van der Waals interactions and capillary forces, forms an insoluble interfacial barrier that minimizes droplet-surface adhesion (*27*).

To quantify droplet-surface adhesion, we measured the CSA (α), defined as the minimum tilt angle at which a droplet initiates sliding (Fig. 1D and Methods and Materials), for protein and pure water droplets on slippery organogel surfaces (movie S1). Droplets were dispensed onto an angle-adjustable platform (Fig. 1E), with protein physicochemical properties detailed in table S1. Initial CSA measurements immediately after droplet deposition showed minimal adhesion, with all droplets sliding at angles near 10°. Notably, after a 20-min resting period, protein-containing droplets exhibited markedly increased CSA values: 26° for lysozyme (Lyz) and 30° for bovine serum albumin (BSA). In contrast, pure water droplets maintained a low CSA of 6°, sliding readily without adhesion buildup. This pronounced time-dependent adhesion enhancement, uniquely observed in protein droplets, underscores the role of progressive protein adsorption at the droplet-surface interface, a phenomenon absent in nonprotein systems.

### Mechanistic insights into time-dependent protein adhesion at solid substrates

To elucidate the time-dependent adhesion mechanism of protein droplets, we focused on BSA as a model protein. To isolate protein-specific effects, we first equilibrated surface tension by preparing a 9% (v/v) ethanol solution (surface tension: 51.3 mN m^−1^) to match that of the BSA solution (52.1 mN m^−1^), thereby eliminating surface tension as a confounding variable (fig. S2, A and B). Comparative analysis of CSAs between BSA and ethanol droplets over time revealed distinct adhesion kinetics, confirming that surface tension alone could not account for the observed behavior (fig. S2C). Droplet volume was limited to several microliters to restrict the influence of gravity on CSA (fig. S3).

**Fig. 2.**
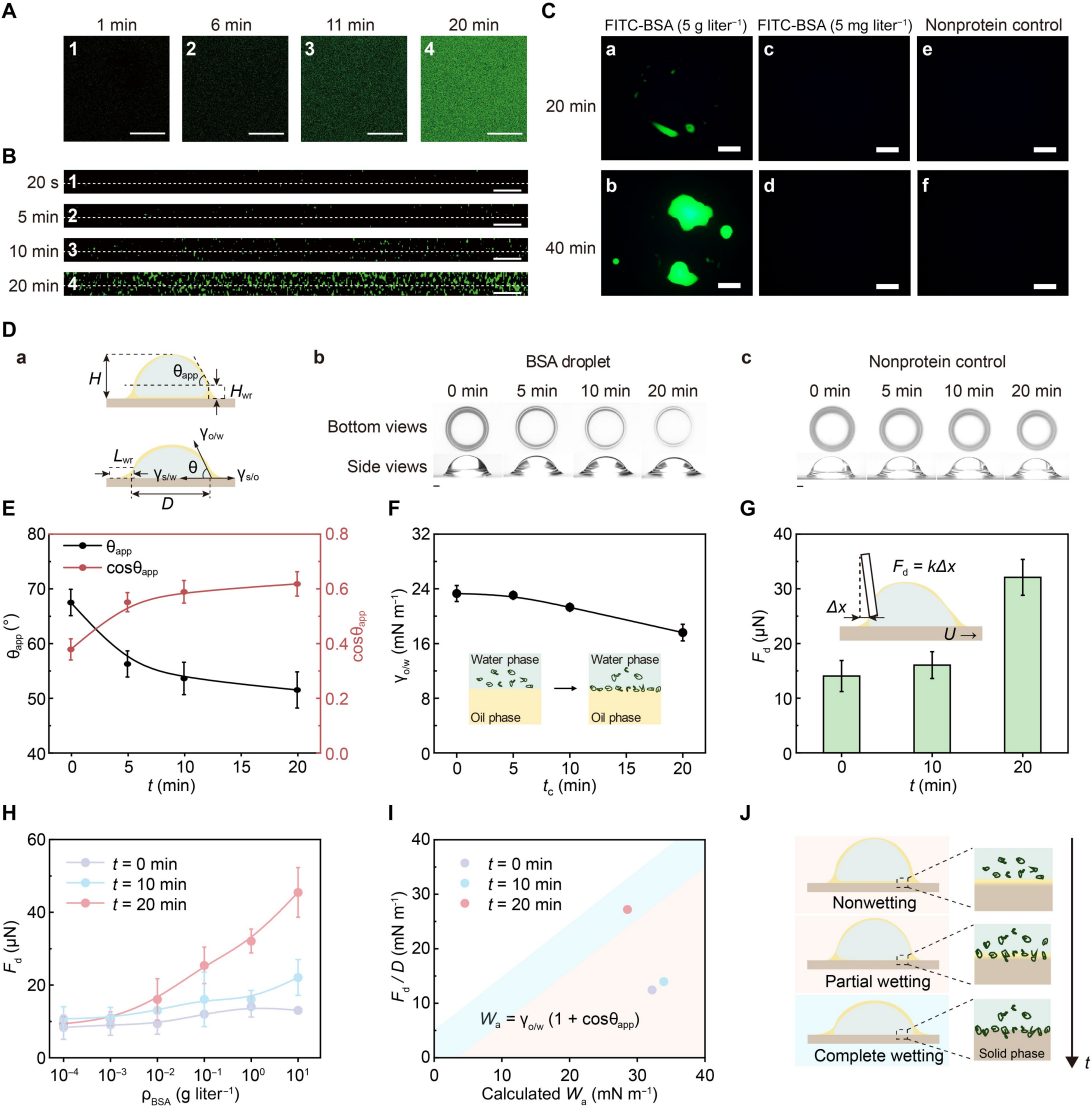
Protein adsorption dynamics and interfacial behavior on slippery organogel surfaces. (**A**) Solid substrate real-time fluorescence profiles of BSA droplet (1 g liter^−1^) under varying rest times, revealing the interfacial protein accumulation process. Scale bar, 100 μm. (**B**) Cross-sectional real-time fluorescence profiles of BSA droplet (1 g liter^−1^) under varying rest times. The dotted line indicates the solid-liquid interface. Scale bar, 20 μm. (**C**) Fluorescence imaging of time- and concentration-dependent protein residual: (a and b) Residual fluorescence of FITC-BSA droplets (5 g liter^−1^) after 20 and 40 min rest; (c and d) 5 mg liter^−1^ FITC-BSA droplets under identical conditions; and (e and f) Pure water nonprotein controls. Scale bar, 500 μm. (**D**) Morphological evolution of droplets under varying rest times (*t* = 0, 5, 10, and 20 min): (a) Schematic defines wetting ridge parameters: length (*L*_wr_), height (*H*_wr_), droplet height (*H*), and base diameter (*D*). (b) BSA droplet (1 g liter^−1^) (bottom/side views). (c) 9% (v/v) EtOH droplet (nonprotein control). Scale bar, 200 μm. (**E**) Quantitative wetting analysis: Time-dependent apparent contact angle (θ_app_) and cosθ_app_ for BSA droplets (1 g liter^−1^). (**F**) Interfacial tension kinetics: Oil-water interfacial tension (γ_o/w_) between BSA (1 g liter^−1^) solution and silicone oil (20 mPa s) as a function of contact time (*t*_c_). (**G**) Adhesion dissipation: Critical dissipative force (*F*_d_) of BSA droplets (1 g liter^−1^) versus rest time. (**H**) Concentration-dependent adhesion: *F*_d_ as a function of BSA mass concentration (ρ_BSA_). (**I**) Adhesion-energy correlation: Normalized dissipative force (*F*_d_/*D*) versus calculated work of adhesion (*W*_a_). (**J**) Protein droplet wetting processes on SLIPS.

To probe protein adsorption dynamics, we employed confocal laser scanning microscopy to track the time-dependent interfacial interactions of fluorescently labeled fluorescein isothiocyanate (FITC)–BSA droplets (1 g liter^−1^) on slippery organogel surfaces. Over a 20-min resting period, BSA droplets exhibited progressive green staining on a solid substrate (bottom view, Fig. 2A), and the side view showed a gradual diffusion of proteins from the droplet to the solid substrate (Fig. 2B).

To directly visualize protein adsorption, fluorescently labeled FITC-BSA droplets (5 g liter^−1^ and 5 mg liter^−1^) were slid off the organogel surface after resting, leaving residual protein traces detectable via fluorescence imaging. Notably, droplets containing FITC-BSA (5 g liter^−1^) exhibited pronounced green fluorescence at rest locations, with signal intensity expanding from 20- to 40-min resting periods (Fig. 2C). In contrast, minimal fluorescence was observed for low-concentration FITC-BSA (5 mg liter^−1^) and pure water, underscoring the concentration- and time-dependent nature of BSA adsorption. Real-time fluorescence imaging further revealed BSA’s dynamic interfacial behavior: proteins initially adsorbed at the water-oil interface and gradually penetrated toward the solid substrate. This progressive interfacial accumulation and substrate contact directly correlated with increased droplet-surface adhesion, driving the observed CSA elevation. Collectively, these findings establish that protein adsorption governs the time-dependent adhesion enhancement unique to protein droplets.

Real-time monitoring of droplet behavior (Fig. 2, D and E) revealed wetting ridge formation, a consequence of interfacial tension equilibrium. Unlike conventional dry surfaces, where evaporation follows a constant-contact-radius mode with fixed three-phase contact lines (*28*), SLIPS exhibited dynamic interfacial evolution. Side-view imaging showed that both BSA and ethanol (EtOH) droplets developed elongated wetting ridges (*L*_wr_) and increased ridge heights (*H*_wr_) over time. However, distinct behaviors emerged: BSA droplets displayed gradual base diameter (*D*) expansion and reduced shadowing intensity, while EtOH droplets shrank proportionally to diameter reduction.

Notably, BSA droplets demonstrated a time-dependent decrease in apparent contact angle ($\theta_{a_{\mathrm{pp}}}$), stabilizing after rest (Fig. 2E), whereas EtOH maintained a constant $\theta_{a_{\mathrm{pp}}}$ of 65 ± 2°. This divergence suggests protein-mediated interfacial restructuring: BSA penetrates the oil layer to anchor at the solid substrate, immobilizing the contact line, whereas EtOH remains stabilized atop the lubricant. These findings collectively support a mechanism where protein adsorption at the water-oil-solid interface drives adhesion enhancement, absent in nonprotein systems.

To unravel the mechanisms governing droplet adhesion, we analyzed the work of adhesion (*W*_a_), the energy required to separate two phases, using the thermodynamic relation: *W*_a_ = γ_s/o_ + γ_o/w_ − γ_s/w_, where γ denotes interfacial tension, and subscripts s, o, and w represent the solid surface, silicone oil, and aqueous droplet, respectively. Direct measurement of γ_s/o_ and γ_s/w_ remains experimentally challenging, prompting us to simplify the framework by integrating Dupré’s equation with Young’s equation (*29*): γ_s/o_ = γ_o/w_ cosθ + γ_s/w_, yielding the Young-Dupré equation: *W*_a_ = γ_o/w_(1 + cosθ). This approach allowed us to bypass direct solid-phase tension measurements while retaining thermodynamic rigor.

Time-resolved interfacial tension γ_o/w_ measurements between BSA (1 g liter^−1^) solutions and silicone oil revealed a progressive decline with increasing contact time (*t*_c_) (Fig. 2F), consistent with protein adsorption kinetics.

To quantify adhesion strength, we measured the critical dissipative force (*F*_d_), the lateral force required to initiate droplet sliding (measured at the moment when the droplet moves), via capillary tube-based force spectroscopy (*30*–*32*) (fig. S4 and table S1). *F*_d_ exhibited a time- and concentration-dependent rise: (i) time dependence, *F*_d_ increased steadily with rest time (Fig. 2G); and (ii) concentration dependence, *F*_d_ scaled monotonically with BSA concentration (ρ_BSA_) (Fig. 2H). These trends confirm that both rest duration and protein concentration synergistically amplify adhesion by promoting interfacial protein accumulation and solid-surface contact.

We hypothesized that the enhanced adhesion of protein droplets to the organogel surface arises from progressive wetting of BSA droplets on the underlying solid substrate. To validate this, we calculated the theoretical work of adhesion (*W*_a_) using the apparent contact angle ($\theta_{a_{\mathrm{pp}}}$) for BSA droplets (1 g liter^−1^) at varying rest times and compared these values with the ratio of critical dissipative force to droplet base diameter *F*_d_/*D*. As BSA droplets rested on the lubricant layer, protein adsorption at the water-oil interface facilitated penetration through the oil phase. This process generated downward pressure, driving the droplet through three distinct wetting stages: nonwetting, partial wetting, and complete wetting (Fig. 2J).

Upon achieving complete solid-surface wetting, the *W*_a_ aligned closely with the experimentally measured *F*_d_/*D* (Fig. 2I). In contrast, *W*_a_ significantly exceeded *F*_d_/D in incomplete wetting regimes, underscoring the critical role of interfacial contact in adhesion dynamics. The convergence of theoretical and empirical data robustly corroborates our hypothesis.

### CSA as a visual detection indicator and its dominant properties

**Fig. 3.**
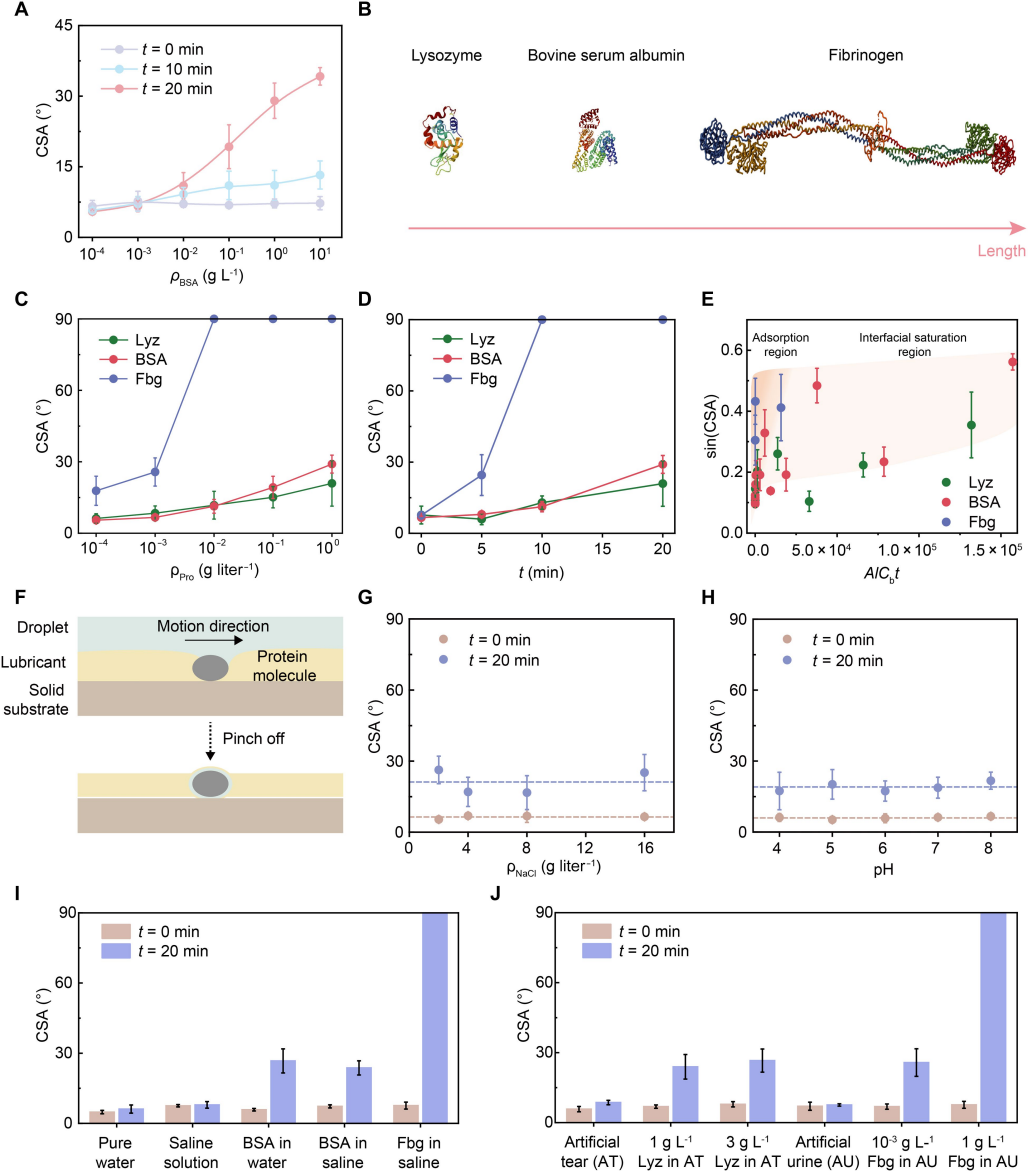
Dominant properties on CSA. (**A**) Protein concentration and rest time: CSA of BSA droplets versus ρ_BSA_ and rest time. (**B**) Structural schematics of Lyz, BSA, and Fbg. (**C**) Protein type: CSA of droplets containing Lyz, BSA, or Fbg, plotted against protein concentration (ρ_pro_). (**D**) Time-dependent CSA measurements for protein droplets (1 g liter^−1^; Lyz/BSA/Fbg) during rest periods. (**E**) The data show a consistent trend for different systematic parameters until the protein saturation on the solid substrate, reflecting a relationship between sin(CSA) and $A{\mathrm{lc}}_{b}t$, $A=\frac{\gamma_{o/w}D^{\prime }}{\rho gd^{2}}$. (**F**) Protein-molecule–induced adhesion enhancement when the droplet begins sliding. (**G**) CSA of Fbg droplets (10^−3^ g liter^−1^) as a function of NaCl concentration (ρ_Na__Cl_). (**H**) CSA of Fbg droplets (10^−3^ g liter^−1^) across varying pH values. (**I**) Comparative CSA analysis for control droplets (pure water, saline) and protein-containing samples [BSA (1 g liter^−1^) in water/saline, Fbg (1 g liter^−1^) in saline] at 0 and 20 min rest times. (**J**) CSA of simulated biological fluids: blank artificial tears (AT) and urine (AU), along with disease-relevant models, AT spiked with Lyz (1 g liter^−1^; dry eye syndrome mimic) or Lyz (3 g liter^−1^; healthy tear control), and AU spiked with Fbg [10^−3^ g liter^−1^; chronic kidney disease (CKD) indicator] or 1 g liter^−1^ Fbg (severe CKD model), measured after 0- and 20-min rest.

We further explored a suitable naked-eye detection indicator. We evaluated the CSA across a broad BSA concentration range (10^−4^ to 10 g liter^−1^) to establish a visual detection threshold (Fig. 3A). The observed monotonic increase in CSA with rising protein concentration, consistent with *F*_a_ trends, validates CSA as a simple, naked-eye indicator for protein detection, bridging fundamental adhesion mechanisms with practical diagnostic applications.

To evaluate suitability for naked-eye detection, we tested the sliding velocity of protein droplets on surfaces (see Supplementary Text and fig. S5) and compared it with the CSA. The CSA was selected as the detection index due to its broader linear range relative to sliding velocity, providing greater sensitivity for visual analysis.

We systematically explored how protein properties affect the CSA of droplets. Three proteins, Lyz, BSA, and fibrinogen (Fbg) (*33*), were tested for CSA (Fig. 3, B and C, and table S2). After a 20-min rest period, Fbg droplets exhibited a sharp increase in CSA, with droplets becoming irreversibly pinned to the surface at concentrations above 0.1 g liter^−1^. In contrast, BSA- and Lyz-containing droplets showed gradual CSA increases, highlighting a hydrophobicity-dependent adhesion response. This behavior was corroborated by time-course CSA measurements at a fixed protein concentration (ρ_pro_ = 1 g liter^−1^), where identical trends emerged across rest intervals (Fig. 3D).

To investigate important properties for CSA increase, we further applied Fick’s first law (*34*, *35*): $J=–D^{\prime }\frac{dc}{dx}$ (where *J* is protein diffusion flux, *D*′ is the protein diffusion rate, $\frac{dc}{dx}$ is the concentration gradient) and molecule adhesion force (see Supplementary Text), and the data show a consistent trend for different systematic parameters in the beginning until the protein saturation on the solid substrate, showing a relationship between the sin(CSA) and $A{\mathrm{lc}}_{b}t$ (Fig. 3, E and F, where $A=\frac{\gamma_{o/w}D^{\prime }}{\rho gd^{2}}$, *l* is the protein molecule length, $c_{b}$ is the protein molar concentration, ρ, *d* is the density and diameter of the protein droplet respectively, *g* is the gravitational constant).

Next, we sought surfaces that exhibit pronounced post-rest CSA variations (figs. S6 to S8) and systematically investigated key influencing factors: surface type (see Supplementary Text and fig. S7), lubricant viscosity (see Supplementary Text and fig. S7), and slippery organogel surface microstructure (see Supplementary Text and fig. S8). These studies revealed that a slippery organogel surface lubricated with 4.5 mPa s silicone oil optimizes CSA contrast, making it ideal for naked-eye protein detection.

To mimic the complex ionic environment of biological fluids, characterized by diverse ion concentrations and pH values, we further investigated the effects of NaCl concentration (ρ_Na__Cl_) and pH on CSA. These parameters were adjusted to reflect physiological ranges, aligning with in vivo fluid compositions. Notably, ionic strength and pH had minimal impact on CSA changes compared to rest time and protein concentration (Fig. 3, G and H). Protein-free controls (pure water) and ionic environment controls (saline) confirmed these observations: droplets containing BSA (1 g liter^−1^) showed no significant CSA difference between water and saline, whereas Fbg at the same concentration markedly enhanced droplet adhesion (Fig. 3I).

Our findings demonstrate the potential to harness protein adhesion dynamics for diagnosing protein-related disorders. For instance, chronic dry eye syndrome is characterized by significantly reduced levels of Lyz and lactoferrin in tear fluid, biomarkers that average ∼3 g liter^−1^ in healthy individuals but fall below 1 g liter^−1^ in affected patients (*36*). Conversely, elevated urinary protein (proteinuria) serves as a hallmark of chronic kidney disease and diabetes, with healthy individuals exhibiting negligible urinary protein levels (*37*).

To validate clinical applicability, we engineered simulated biofluids, artificial tears and urine, methodically spiked with disease-relevant proteins: Lyz (dry eye biomarker) and Fbg (renal dysfunction indicator) (*38*) (Fig. 3J). Remarkably, droplets mimicking pathological conditions exhibited substantial CSA increases after a 20-min rest, mirroring the adhesion enhancement observed in native biofluids. This responsiveness underscores our platform’s capacity to detect clinically relevant protein thresholds through simple, time-dependent CSA measurements.

### CSA-driven proteinuria screening and monitoring: A paradigm shift in noninvasive diagnostics

**Fig. 4.**
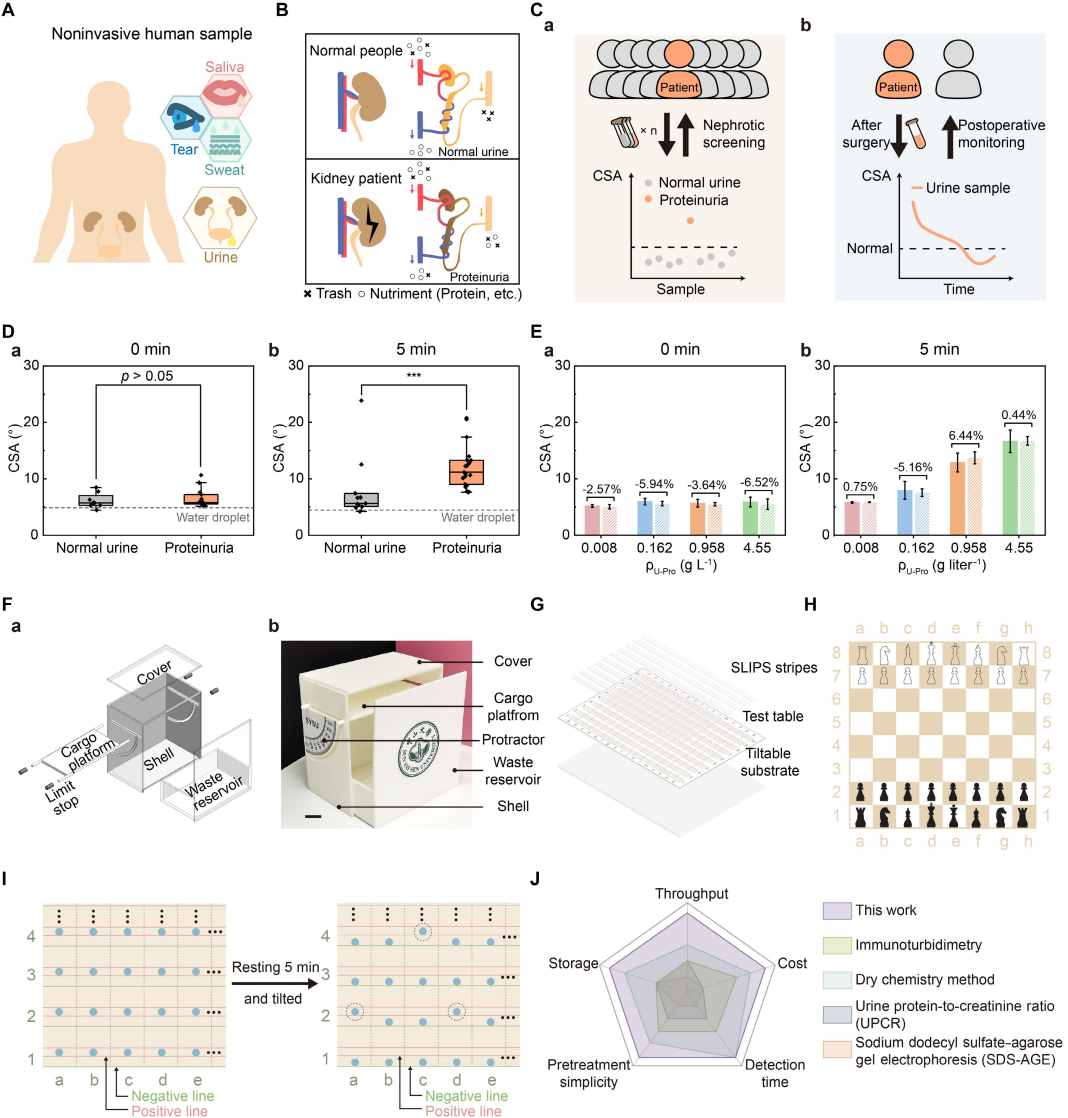
Naked-eye detection via CSA. (**A**) Noninvasive biofluid sampling: Schematic of CSA-based protein detection in urine, tears, saliva, and sweat. (**B**) Schematic diagram of normal urine and proteinuria. (**C**) Clinical application workflow: (a) Diagnostic threshold: normal urine (low CSA) versus proteinuria (high CSA). Population-level nephropathy screening using high-CSA proteinuria as a visual indicator. (b) Postoperative monitoring: Longitudinal urine CSA tracking; recovery is signaled by CSA returning below the 0.15 g liter^−1^ threshold (dashed line). (**D**) CSA diagnostic accuracy: (a) CSA values of clinical urine samples without resting, showing the necessity of droplet rest. (b) CSA values of clinical urine samples after 5-min rest (***P < 0.001 versus controls, the presence of outliers in normal samples can be reasonably attributed to minor contamination during sampling). (**E**) Pretreatment-free verification: The CSA values of noncentrifuged (solid) and centrifuged (striped) urine samples with rest time of (a) 0 and (b) 5 min. The labeled percentage change of the centrifuged sample from the noncentrifuged sample showed that the difference was limited. (**F**) Portable CSA device: Prototype for home-based POC. Scale bar, 2 cm. (**G**) Mass screening device: Schematic diagram for mass screening. (**H**) Chessboard diagram: Design inspiration for a mass screening device. (**I**) Mass screening operation processes: First, the sample droplets are deposited between the two positive lines (red, left). After a 5-min rest, the device is tilted to observe droplet sliding behavior (right). If the droplet remains stationary, it indicates the presence of the protein. Conversely, if the droplet slides to the negative line (green), it suggests the absence or minimal presence of the protein. (**J**) Radar comparison of the CSA-indicated protein detection methods with representative methods for proteinuria detection. (A) to (C) were created in BioRender. X. Han (2026) https://BioRender.com/p344iqx.

The shift toward noninvasive diagnostics has driven the adoption of biofluids such as urine, tears, sweat, and saliva, which provide rich biological insights while eliminating risks tied to invasive procedures (Fig. 4A). These samples enable frequent collection, making them ideal for large-scale screenings and longitudinal health monitoring, a critical advantage for managing chronic conditions like kidney disease.

Our results demonstrate that CSA serves as a robust, quantitative proxy for urinary protein concentration, bridging the gap between laboratory findings and clinical utility. By correlating CSA with proteinuria (Fig. 4B), a hallmark of renal dysfunction, we establish a direct visual metric to distinguish healthy urine (low CSA) from pathological samples (elevated CSA).

### Scalable clinical applications: Population-level screening

Elevated CSA enables rapid identification of proteinuria in nephropathy patients, streamlining mass screenings through simplified workflows and high-throughput compatibility (Fig. 4Ca); postoperative monitoring—longitudinal CSA tracking in chronic kidney disease patients offers a dynamic recovery metric; sustained CSA values below the 0.15 g liter^−1^ threshold signal restored renal function (Fig. 4Cb). This approach minimizes procedural complexity, eliminates costly instrumentation, and prioritizes patient comfort—key advancements for equitable healthcare delivery.

### Validation of the detection method and POCT device

To validate the efficacy of the developed assay, clinical urine samples were collected and analyzed (urine protein concentration denoted as ρ_U-pro_; Fig. 4D). A 4-μl aliquot of each sample was applied to the organogel surface, and the CSA was measured after a defined rest period. Optimization of rest time revealed that distinct CSA differences between proteinuria and normal urine emerged within minutes (Fig. 4Db, number of normal urine and proteinuria samples is 11 and 21, respectively, tested with the turbidimetric method), demonstrating rapid detectability. Notably, no significant discrepancies were observed between noncentrifuged and centrifuged samples (Fig. 4E), confirming that the method eliminates the need for sample pretreatment, streamlining the workflow for practical use.

This urine protein detection approach relies solely on the intrinsic physical adsorption of proteins to the surface, translating microscopic protein-surface interactions into observable macroscopic CSA variations. By circumventing the need for complex signal transduction or labeling, the technique offers a simplified, pretreatment-free solution highly suitable for proteinuria screening and longitudinal monitoring.

To enable decentralized testing, the POCT device was designed for home-based self-testing, containing a cover, a shell, a tiltable cargo platform, a protractor for CSA readouts, and a waste reservoir (Fig. 4F and fig. S9), with storage and temperature/humidity stability (figs. S10 and S11) and large-scale disease screening (Fig. 4, G to I, and fig. S12) of biological fluid samples with a cost as low as $0.80 per 100 tests (table S3), offering crucial advantages compared with conventional detection methods (Fig. 4J). This innovation underscores its potential for affordable, scalable implementation in resource-limited settings or routine clinical care, bridging the gap between laboratory-based diagnostics and accessible, real-time health monitoring.

## DISCUSSION

This study explores the influence of rest time, protein concentration, and protein type on the CSA of protein droplets on SLIPSs, a class of lubricant-impregnated materials known for their unique wetting properties. Our key observation, a marked increase in CSA for protein-laden droplets relative to pure water controls after rest, is rooted in the dynamic interplay of protein adsorption at the water-oil interface and subsequent droplet-solid surface interactions. As proteins accumulate at the interface during the rest period, they modify the interfacial energy and adhesive forces between the droplet and the SLIPS, leading to measurable changes in CSA. This mechanism contrasts with purely hydrodynamic effects, such as those governing sliding velocity, and underscores the role of surface chemistry in modulating droplet behavior.

Building on this mechanistic understanding, we introduce a label-free, pretreatment-free diagnostic approach that leverages the intrinsic physical adsorption of proteins to translate nanoscale surface interactions into macroscale CSA readouts. Unlike conventional protein detection methods, which often rely on complex biochemical labeling, antibody-based assays, or time-consuming sample preparation, our strategy eliminates the need for signal amplification or specialized reagents. By directly linking protein concentration and adsorption kinetics to observable changes in droplet sliding behavior, the method streamlines the diagnostic workflow, making it accessible for point-of-care applications. Crucially, the absence of pretreatment, evidenced by consistent CSA measurements in both centrifuged and noncentrifuged clinical samples, further enhances its practicality, reducing operational complexity and minimizing user error.

The utility of this approach is particularly pronounced in proteinuria screening, where early detection of abnormal urinary protein levels is critical for managing chronic kidney disease, diabetes, and other protein-related disorders. By demonstrating that CSA can distinguish between proteinuria and normal urine within minutes (Fig. 4D) and across a physiologically relevant concentration range, we validate its potential as a rapid, visual diagnostic tool. The required surface size is as small as several square centimeters, reducing the material cost and improving the parallel testing capability. The ultralow cost, high throughput (170 samples tested in parallel in 5 min on an A4-size substrate), and naked-eye interpretability of CSA measurements address key barriers to widespread screening, especially in resource-limited settings or for longitudinal home monitoring. These features align with the growing demand for minimally invasive, patient-centric diagnostics, where frequent sampling and real-time feedback improve adherence and clinical outcomes.

Beyond proteinuria, this work establishes a foundational framework for using SLIPS to probe protein-surface interactions in diverse biological fluids, including tears, saliva, and sweat. The ability to visualize subtle changes in droplet adhesion through CSA provides a versatile platform for studying other protein biomarkers associated with diseases like chronic dry eye syndrome or cardiovascular disorders. By integrating this technology into compact, user-friendly POCT devices (Fig. 4, F to I), we bridge the gap between laboratory-based analytics and accessible, on-demand health monitoring, positioning it as a transformative tool for both clinical and community-based applications.

In summary, our study transcends traditional diagnostic paradigms by harnessing the physics of droplet wetting and protein adsorption to create a simple, cost-effective, and scalable method for protein detection. By emphasizing macroscopic observables over microscopic complexity, we offer a robust solution to unmet needs in early-stage disease screening and continuous patient management, highlighting the power of materials science to drive innovation in translational medicine.

## MATERIALS AND METHODS

### Fabrication of organogel surface

Polydimethylsiloxane oligomers were mixed with a cross-linker in a ratio of 10:1 (w/w). After thorough stirring and mixing, the mixture was added to the substrate and spin-coated at 300 rpm for 15 s and then heated at 80°C for 60 min in a vacuum-drying oven. Subsequently, to obtain the organogel surface, the PDMS was peeled off from the substrate, then immersed in silicone oil for 24 hours, and then placed on a substrate vertically to remove excess silicone oil.

### Fabrication of nanostructure-based SLIPS

A commercial spray Glaco was used to generate a nanostructure on the PDMS surface. PDMS was then immersed in silicone oil and placed on a substrate vertically to remove excess silicone oil.

### Fabrication of liquid brush surface

The aluminum plate was treated with plasma for 60 s, and then immersed in HMS-991 and dried at room temperature for 48 hours.

### CSA measurement

The droplets were first released on the horizontal surface, and after different rest times, the surface slowly tilted to allow the droplets to slide off the surface with a tilting velocity of about 1° s^−1^. The sliding process was recorded using a high-speed camera, after which the images were analyzed by the software ImageJ.

### Critical dissipative force measurement

The method of measuring the dissipative force (*F*_d_) involves observing the deflection of a capillary tube that is inserted into the droplet placed on the surface, which is placed on a movable platform (*30*, *31*). As the platform moved, the droplet remained static due to the capillary tube. The amount of deflection of the capillary could reflect the dissipative force.

### Characterizations

The surface/interfacial tensions were measured at room temperature (22° ± 1°C) with an automatic surface tension meter (JK99, POWEREACH). The droplet morphology was recorded simultaneously by a high-speed camera and a contact angle meter (JC2000C1, POWEREACH). For fluorescence imaging of the protein, the droplets slid off the surface, and the locations of the droplets on the surfaces were imaged on a fluorescence inverted microscope. A 480-nm laser was chosen for the excitation of fluorescein isothiocyanate, and the emission was collected at 500 to 540 nm. The confocal laser scanning microscope (NIKON AX, Japan) was used for Z-stack imaging. NIS-Element Viewer V5.21 Image software (NIKON) was used to analyze the fluorescence images.
