# Supplementary material for: Interfacial amplifier: High-throughput visual protein detection via droplet dynamics
Source: Sci Adv. 2026 Aug 12;12(33):eaef6559. doi: 10.1126/sciadv.aef6559 (PMC13464474; doi:10.1126/sciadv.aef6559)
Supplement: Supplementary file 1 [file sciadv.aef6559_sm.pdf]

Science Advances
AAAS

Supplementary Materials for

Interfacial amplifier: High-throughput visual protein detection via droplet dynamics

Tianxin Ge *et al.*

Corresponding author: Liqiu Wang, liqiu.wang@polyu.edu.hk; Zong Dai, daizong@mail.sysu.edu.cn; Xing Han, hanx83@mail.sysu.edu.cn

Sci. Adv. **12**, eaef6559 (2026)
DOI: 10.1126/sciadv.aef6559

The PDF file includes:

Figs. S1 to S12
Supplementary Text
Tables S1 to S3
Legend for movie S1

Other Supplementary Material for this manuscript includes the following:

Movie S1

Supplementary Text and Figures

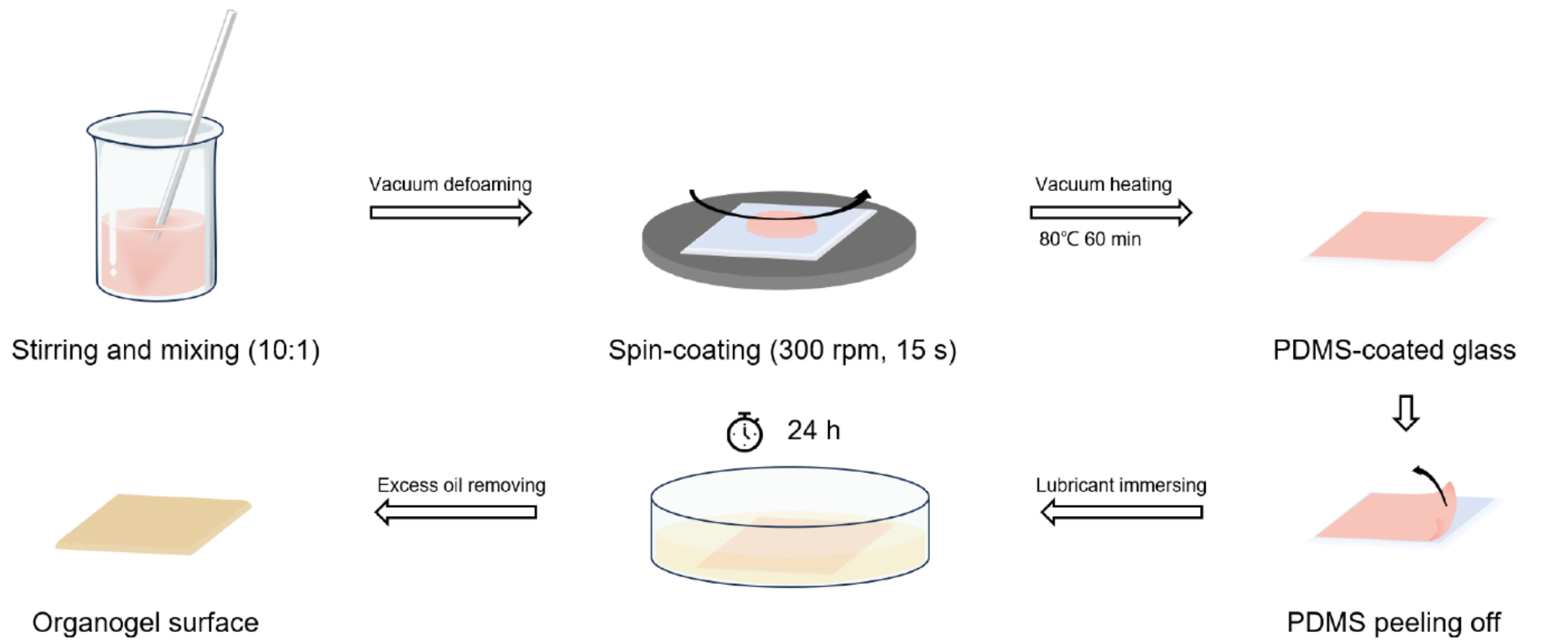

Fig. S1. Fabrication of the slippery organogel surface. Polydimethylsiloxane oligomers were mixed with a cross-linker in a ratio of 10:1 (w/w). After thoroughly stirring and mixing, the mixture was added to the substrate surface using a syringe. After the mixture had been spread evenly on the substrate surface, it was placed on a glass slide by the spin-coating method at 300 rpm for 15 s and was then heated at 80 ℃ for 60 min in a vacuum-drying oven. Subsequently, to obtain the organogel surface, the PDMS was peeled off from the glass, then immersed in silicone oil for 24 h, and placed on a substrate vertically to remove excess silicone oil.

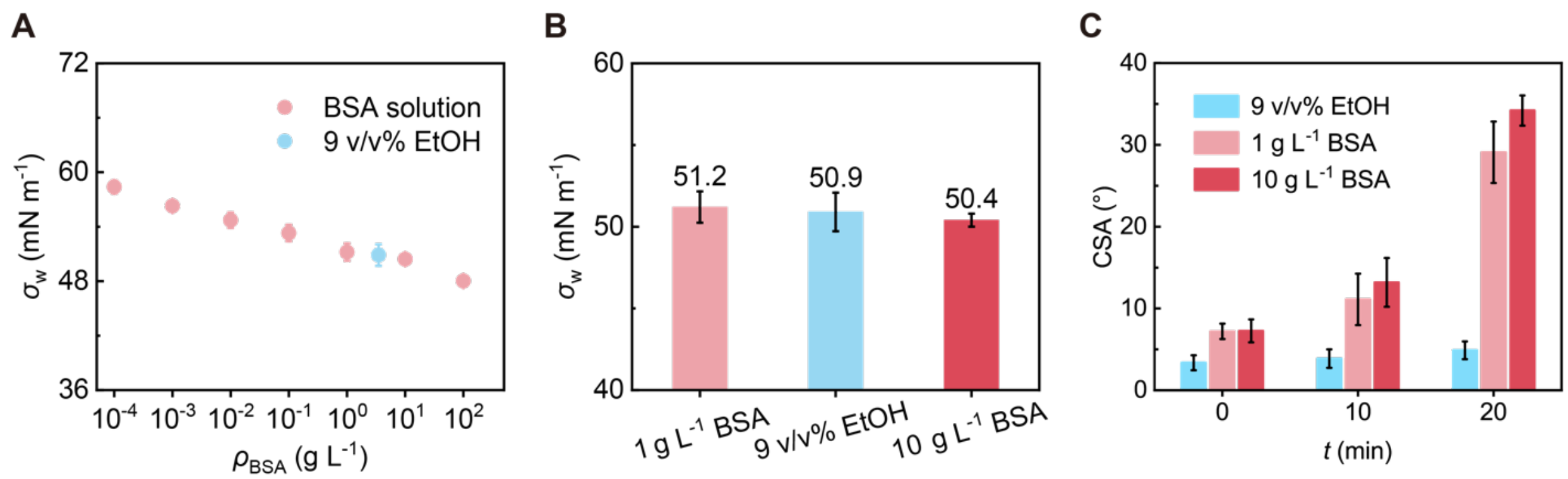

Fig. S2. Surface tension measurement. (A) The surface tension of BSA droplet and 9 v/v% EtOH solutions. (B) The surface tensions of 1, 10 g L^{-1}, and 9 v/v% EtOH solutions. (C) The comparison of CSA with droplets containing 1 and 10 g L^{-1} BSA, and 9 v/v% EtOH solution after 0, 10, and 20 min rest.

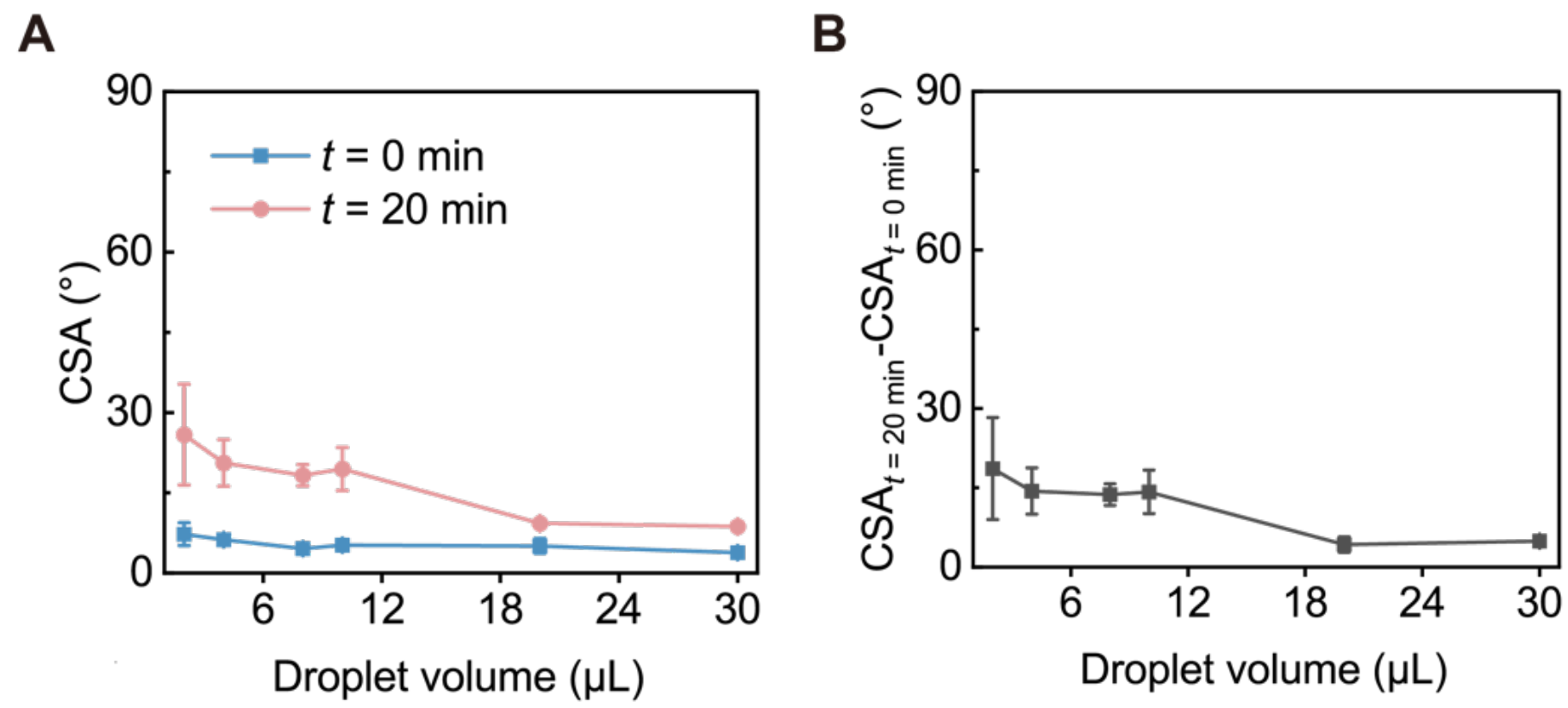

Fig. S3. Variation of (A) CSA and (B) the difference in CSA between resting and unresting samples, plotted as a function of BSA droplet volume. As the droplet volume increases, gravitational forces become more dominant relative to interfacial adhesion between the droplet and the surface, which in turn reduces the increase in CSA observed under resting conditions.

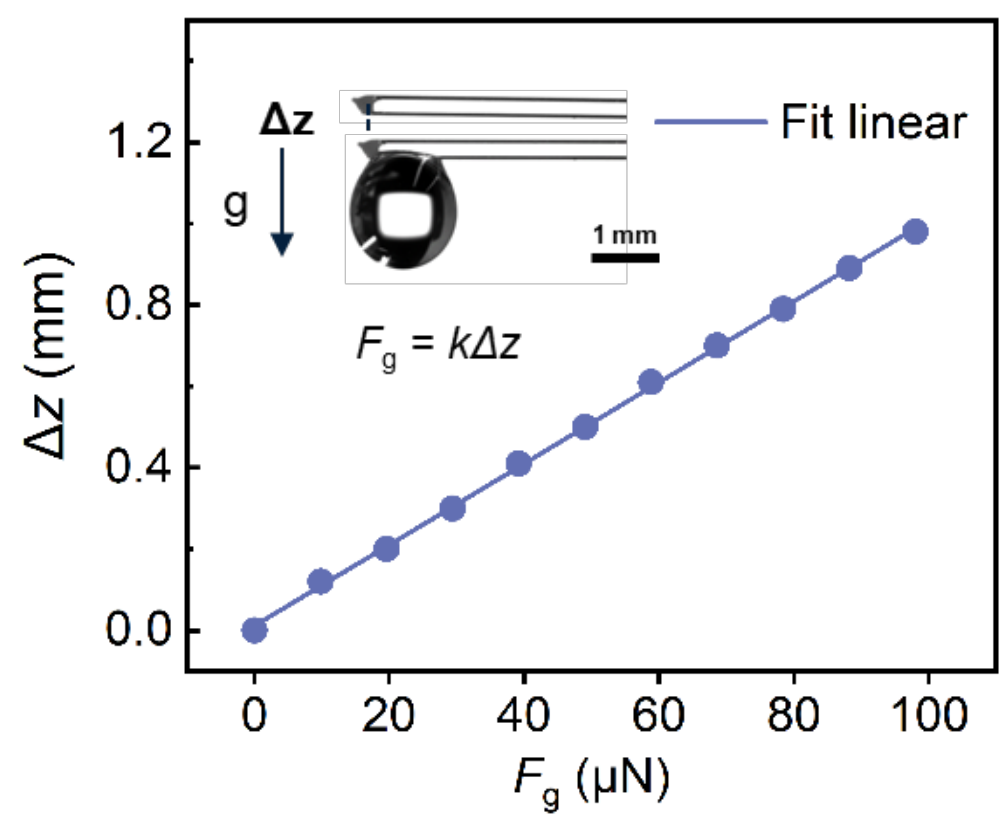

Fig. S4. ***F_d* measurement via cantilever-based force sensor.** F_d is linearly proportional to the deflection of the capillary tube. This can be inferred from the capillary deflection Δx as $F_d = k\Delta x$ where k is the effective spring constant. k can be determined experimentally by placing droplets of different sizes and measuring the capillary deflection Δz due to the droplet weight F_g.

Comparison between sliding velocity and CSA for visual detection

The sliding distances of pure water, 10^{-4} and 10^{2} g L^{-1} BSA droplets within the same time were tested (Fig. S5A). It was observed that the sliding distances of pure water and 10^{-4} g L^{-1} BSA droplets were close, being much larger than that of 10^{2} g L^{-1} BSA droplets. Instantaneous velocity and average velocity were observed to be close. BSA droplets with different concentrations of BSA were used for the average velocity test (Fig. S5B). When the BSA concentration increased from 10^{-3} g L^{-1} to 1 g L^{-1}, the velocity of the droplets decreased steadily with a correlation coefficient of 0.991 and a dynamic range of 10^{3}-fold, and then tended to stabilize. However, the CSA after 20-min rest was obtained with BSA concentrations from 10^{-3} g L^{-1} to 10 g L^{-1} with a correlation coefficient of 0.994 and a dynamic range of 10^{4}-fold (Fig. S8I). For this reason, we chose CSA as our visual detection index.

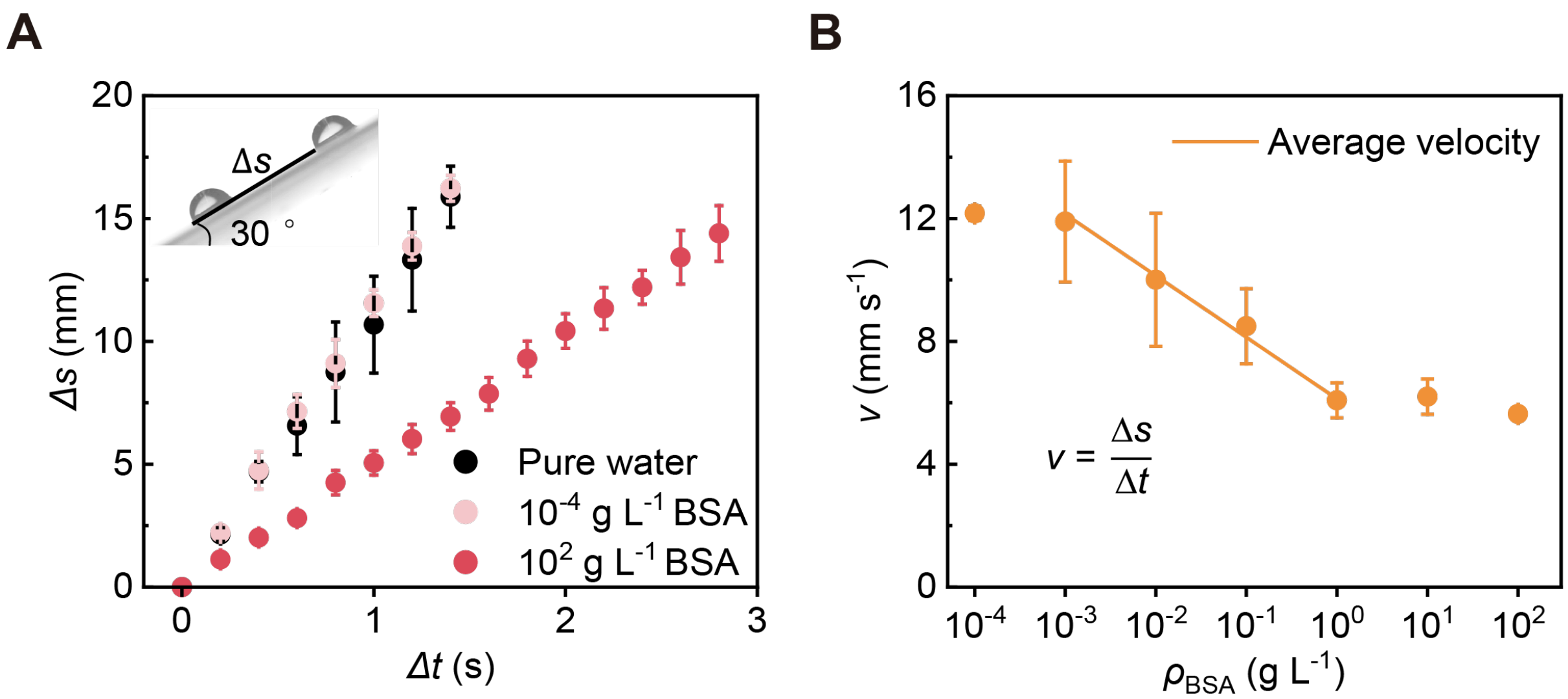

Fig. S5. Sliding velocity of droplets on the surface. (A) The sliding distance Δs of pure water, 10^{-4} and 10^{2} g L^{-1} BSA droplets as a function of sliding time Δt. (B) The average velocity v of the BSA droplets as a function of BSA concentration. The fitting linear with a correlation coefficient of 0.991 and a dynamic range of 10^{3}-fold.

Diffusion-wetting process

For the protein diffusion process analysis, we applied Fick's first law (*34, 35*):

$$J = -D'\frac{\mathrm{d}c}{\mathrm{d}x}, \qquad \text{(Eq. S1)}$$

where J is the protein diffusion flux in the droplet, $\frac{\mathrm{d}c}{\mathrm{d}x}$ is the concentration gradient, D' is the protein diffusion rate, $D' = \frac{K_B T}{6\pi\eta r}$, where K_B is the Boltzmann constant, T is the thermodynamic temperature, η is the droplet viscosity, and r is the water molecular radius. Therefore,

$$J \sim D'\frac{c_b}{d}, \qquad \text{(Eq. S2)}$$

where c_b is protein molar concentration, d is droplet diameter, under the assumption that the interfacial molar concentration $c^* = 0$. To simplify the model, the diffusion of protein in the lubricant is neglected, considering the extremely thin nature of the lubricant between the protein droplet and the solid substrate. The number of protein molecules on the solid substrate N can be described as $N \sim Jtd^2 \sim dD'c_b t$, where N is the protein molecular number. Though balancing the forces on the protein droplet at the critical sliding moment, $mg\sin(\mathrm{CSA}) = FN$, where m is the protein droplet mass, g is the gravitational constant, F is the force due to the protein adhesion for single protein molecule, $F = l\gamma_{\mathrm{o/w}}$, where l is the protein molecule length, neglecting drag force acting on the droplet due to the lubricant. Therefore, the sin (CSA) can be described as

$$\sin(\mathrm{CSA}) \sim Alc_b t, \qquad \text{(Eq. S3)}$$

where $A = \frac{\gamma_{\mathrm{o/w}} D'}{\rho g d^2}$, ρ is the density of the protein droplet.

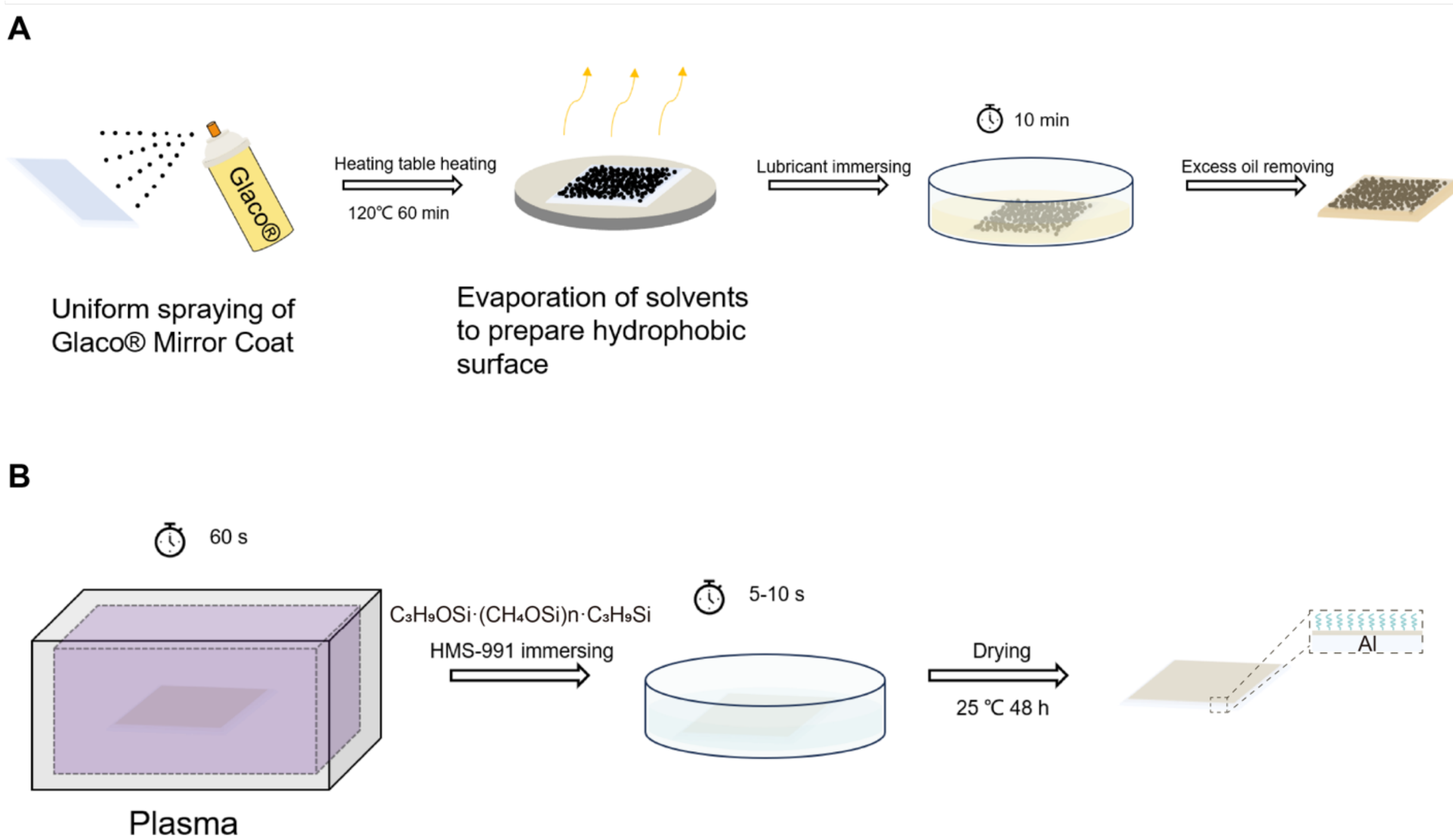

Fig. S6. Fabrication of super-repellent surfaces. (A) The synthesis route of nanostructure-based SLIPS. A commercial spray Glaco (silanized nanoparticles) was used to generate a nanostructure on the PDMS surface. PDMS was then peeled off from the glass and immersed in silicone oil, and placed on a substrate vertically to remove excess silicone oil. In the preparation of micro-nanostructure-based SLIPS, the glass was replaced with a silicon micropillar surface. (B) The synthesis route of the liquid brush surface. The aluminum plate was treated with plasma for 60 s, and then immersed in HMS-991 (Polymethylhydrosiloxane) and dried at room temperature for 48 h.

The CSA on different surfaces

We prepared an array of surfaces with different surface structures (from molecule scale to micro-nanoscale) and conducted CSA test to evaluate surfaces that would exhibit significant differences in CSA after droplet rest, including polydimethylsiloxane (PDMS) surface (smooth surface), micro-nanostructure-based SLIPS (MNS, silicon surface with etched micro-pillar structure and sprayed nanoparticles, representing the SLIPS with two-tier solid structure), liquid brush-treated surface (LB, the surface with molecule layer), nanostructure-based SLIPS (NS, the SLIPS with nanostructure), and organogel surface (OS) (Fig. S7A).

Both BSA and pure water droplets show a CSA of 90° on bare PDMS at both 0 min and 20 min, with no change over time. This results from the poor liquid-repellent ability of the untreated PDMS surface, rendering it unsuitable for protein detection. Droplets all slip easily on other super-repellent surfaces directly at 0 min, exhibiting a low CSA (~5°). Comparatively, after 20 min of resting, the CSAs of the BSA droplets increase, whereas the CSA of the pure water remains unchanged. Among them, BSA droplets have the lowest CSA on MNS (6.8 ± 0.55 °), followed by NS (11 ± 0.33 °) and LB (15 ± 0.39 °), and have the highest CSA on OS (23 ± 3.5 °). The superior liquid repellency of the MNS, NS, and LB surfaces renders them unsuitable for CSA-based protein detection. Their micro-/nano- or molecular-scale structures reduce protein interfacial adsorption and adhesion forces, which inhibits CSA increase. In contrast, the organogel surface has moderate liquid repellency, enabling a clear CSA difference after droplet resting and thus making it suitable for visual protein detection.

The influence of lubricant viscosity on CSA

Noting that the BSA droplets had the largest CSA difference on the organogel surface after droplet rest, we further investigated the suitable viscosity of the lubricant by testing the CSA under two conditions: resting 0 min and 20 min for 1 g L^{-1} BSA droplets; 0 min and 20 min for pure water on organogel surfaces immersed in silicone oil with viscosities of 0.60, 4.5, 20, and 100 mPa s (Fig. S7B). Pure water droplets can easily slide off either directly or after resting, but the increase in the viscosity of silicone oil will cause a slight rise in CSA. As for the BSA droplets, they exhibited easy sliding off, yet a significant increase in the CSA was observed after a 20-min rest, reaching a maximum at a lubricant viscosity of 4.5 mPa s.

To better quantify the change of CSA after resting, we here defined the initial CSA of BSA droplets as CSA_o, the CSA after 20-min rest as CSA_r, and the CSA after 20-min rest of pure water as CSA_w, and introduced the parameters $n_1 = CSA_r/CSA_o$ and $n_2 = (CSA_r - CSA_w)/\rho_{Pro}$ (Fig. S7Ca-b). For naked-eye detection, if a larger value of n_1 means that the detection system can capture a significant change, meaning higher sensitivity. In addition, in medical testing, a large n_1 and n_2 change help accurate diagnose for disease or a pathological condition, reducing the possibility of misdiagnosis. For SLIPS, the ability to detect significant n_1 and n_2 helped to more accurately adjust control parameters, such as the viscosity or type of lubricant, to achieve the desired performance criteria (Fig. S7Da-b).

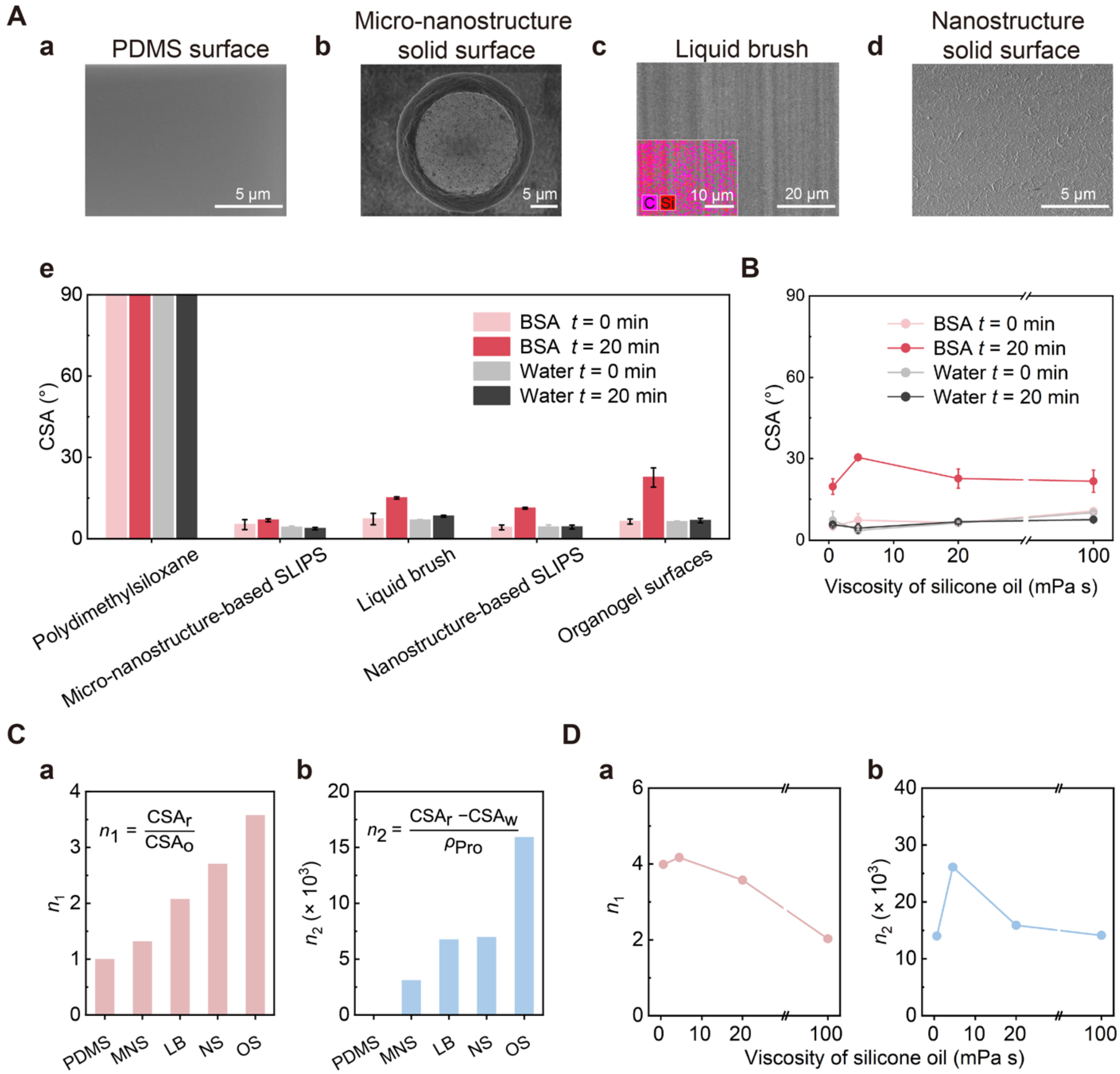

Fig. S7. CSA of BSA and water droplets on different surfaces. (A) (a-d) Scanning electron microscope (SEM) images of the PDMS surface, micro-nanostructure solid surface, LB, nanostructure solid surface, respectively, and (e) CSA of different surfaces after resting 0/20 min. The surfaces included Polydimethylsiloxane (PDMS), Micro-nanostructure-based SLIPS (MNS), Liquid brush (LB), Nanostructure-based SLIPS (NS), and Organogel surfaces (OS). (B) The influence of lubricant viscosity on CSA. (C) The defined parameters for the detection of different surfaces were (a) $n_1 = CSA_r/CSA_o$ and (b) $n_2 = (CSA_r - CSA_w)/\rho_{pro}$. The initial CSA of the BSA droplet is CSA_o, the CSA after resting 20 min is CSA_r, and the CSA of pure water after resting 20 min is CSA_w. The ρ_{pro} was 1 g L^{-1}. (D) The defined parameters for the detection of organogel surfaces immersed in silicone oil with viscosities of 0.60, 4.5, 20, and 100 mPa s: (a) n_1 and (b) n_2.

The influence of the microstructures of organogel surface on CSA

We prepared the micro-pillar SLIPS and conducted CSA test to compare them with smooth SLIPS to investigate the influence of surface microstructure on detection performance. We tested the CSA of the 1 g L^{-1} BSA droplets on micro-pillar SLIPS with different rest times (Fig. S8). Upon comparison with the organogel surface, the CSA of 1 g L^{-1} BSA droplets exhibited a significant increase following 20-min rest for pitch = 20 μm.

The results can be explained by applying Cassie–Baxter model on SLIPS. For the surface with microstructure, the modified contact angle θ^* is related to the solid fraction f, $\cos\theta^* = f\cos\theta + f - 1$. When the pillar array is dense, the BSA droplet would penetrate the structure, forming Wenzel state on the slippery surface, where $f > 1$, causing the modified work of adhesion $W_a^* = \gamma_{o/w}(1 + \cos\theta^*) > W_a$ and increasing the CSA. When the pillar array is sparse, the droplet would be Cassie state, reducing the solid fraction and modified work of adhesion. The CSA would be slightly reduced (Fig. S8G).

Subsequently, we assessed the CSA of BSA droplets after 10-min rest on micro-pillar SLIPS (Fig. S8I). As the BSA concentration escalated from 10^{-3} g L^{-1} to 10^{-1} g L^{-1}, there was a sharp rise in the CSA of the droplet, which subsequently oscillated around 43.3°. The linear correlation coefficient was determined to be 0.999, with a narrow dynamic range of only 10^2-fold. In contrast, after 10-min rest on the organogel surface, the CSA was obtained at BSA concentrations ranging from 10^{-4} to 10 g L^{-1} with a correlation coefficient of 0.990 and a dynamic range of 10^6-fold. Furthermore, the CSA values obtained after resting for 20 min covered BSA concentrations ranging from 10^{-3} to 10 g L^{-1}, with a correlation coefficient of 0.994 and a dynamic range of 10^4-fold. The organogel surface exhibited a wider detection range, representing the optimal configuration for protein naked-eye detection.

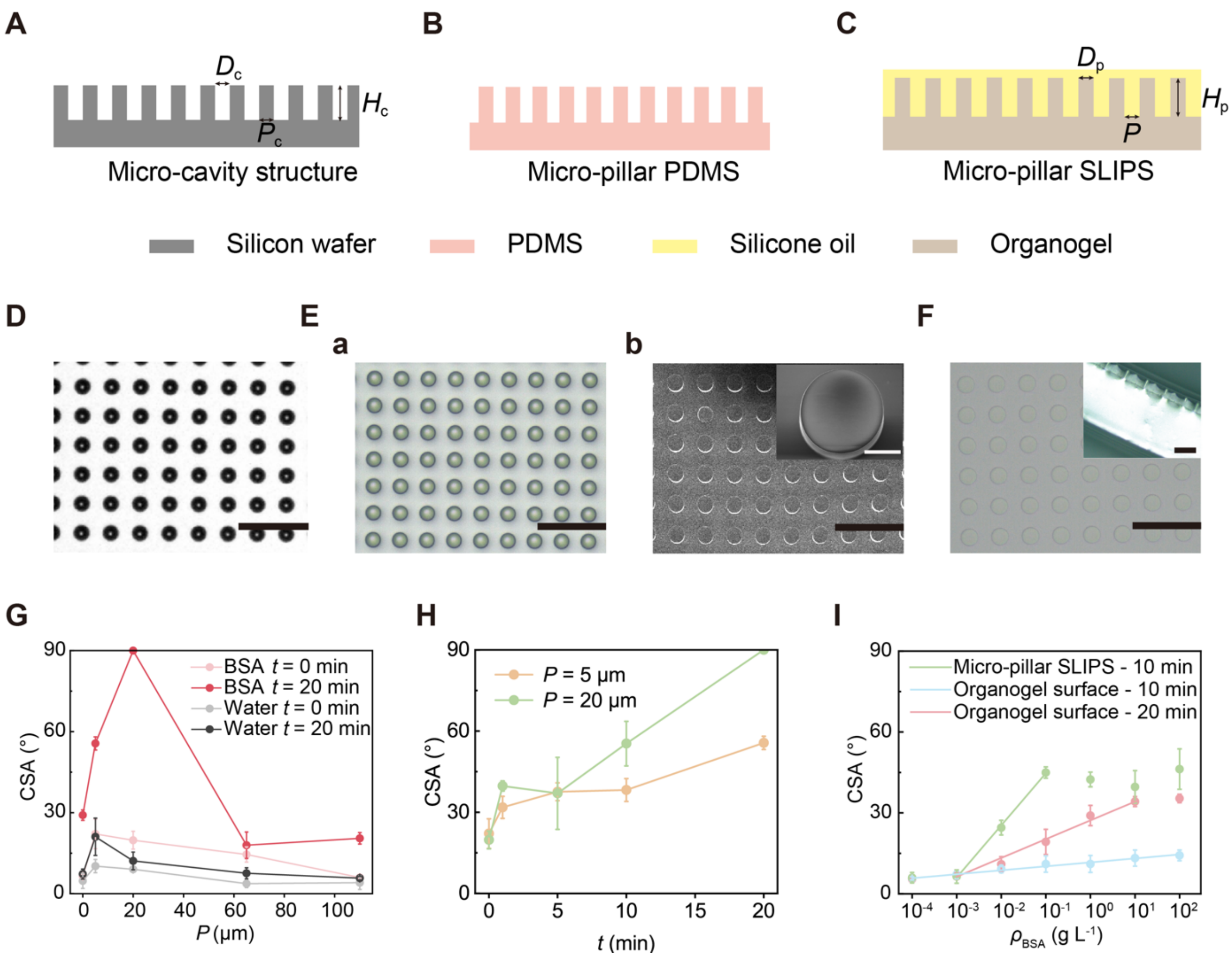

Fig. S8. The CSA on micro-pillar SLIPS. (A) Schematic image of micro-cavity structure. The dimension was referred to as the cavity distance P_c, cavity height H_c and diameter of cavity D_c. P_c = 5/20/60/100 μm, H_c = 40 μm, D_c = 20 μm. (B) Schematic image of cylindrical micro-pillar PDMS. (C) Schematic image of micro-pillar SLIPS. The dimension was referred to as the pillar distance P, pillar height H_p, and diameter of pillar D_p. (D) Top-view optical image of the silicon micro-cavity structure fabricated via silicon etching, scale bar: 100 μm. (E) Top-view (a) optical image and (b) SEM images of the micro-pillar PDMS templated from the cavity structure, scale bar: 100 μm, and 10 μm for the insert image in (b). (F) Top-view and side-view optical images of the micro-pillar SLIPS formed by infusing lubricant oil into the cylindrical micro-pillar PDMS, scale bar: 100 μm. After swelling, D_p and H_p reached 25 μm and 48 μm, respectively. P reached 5 μm, 20 μm, 65 μm, and 110 μm, respectively. (G) The variation of CSA on micro-pillar SLIPS as a function of pillar distance for 1 g L^{-1} BSA droplet and pure water droplet. (H) The variation of CSA on micro-pillar SLIPS as a function of rest time for 1 g L^{-1} BSA droplet. (I) The variation of CSA on micro-pillar SLIPS as a function of BSA concentrations. The linear fitting of BSA droplets resting for 10 min on micro-pillar SLIPS ($y = 19.3 \lg x + 64.0$), resting for 10 min on organogel surface ($y = 1.47 \lg x + 11.7$), and resting for 20 min on organogel surface ($y = 6.96 \lg x + 27.2$).

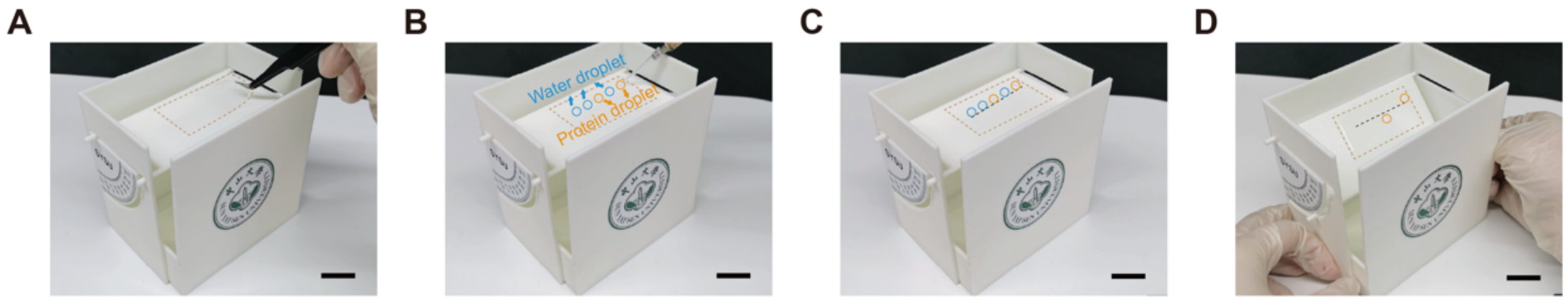

Fig. S9. Portable CSA device operation processes: (A) Organogel surface alignment; (B) droplet deposition; (C) 5-min rest; (D) tilt-induced sliding with CSA measurement. Scale bar: 2 mm.

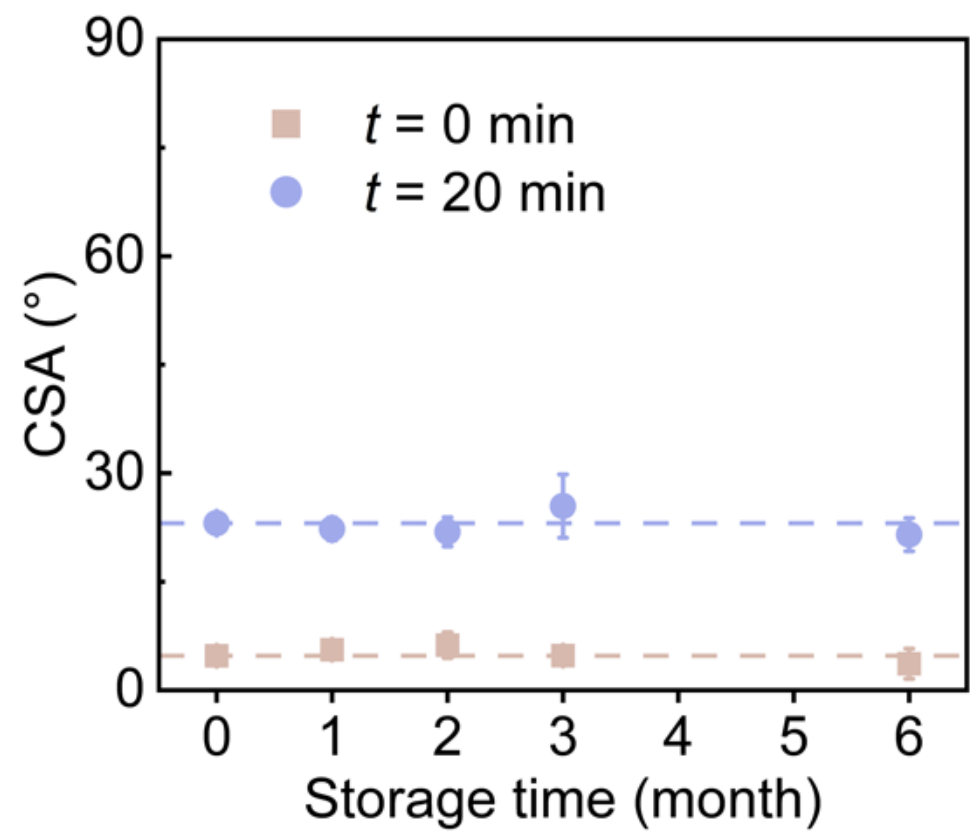

Fig. S10. Storage stability of organogel surface. The CSA of the BSA droplet shows limited variation for rest/unrest samples after months of storage, showing the storage stability of the organogel surface. Storage method: the surfaces were immersed in silicone oil and stored in sealed petri dishes at room temperature.

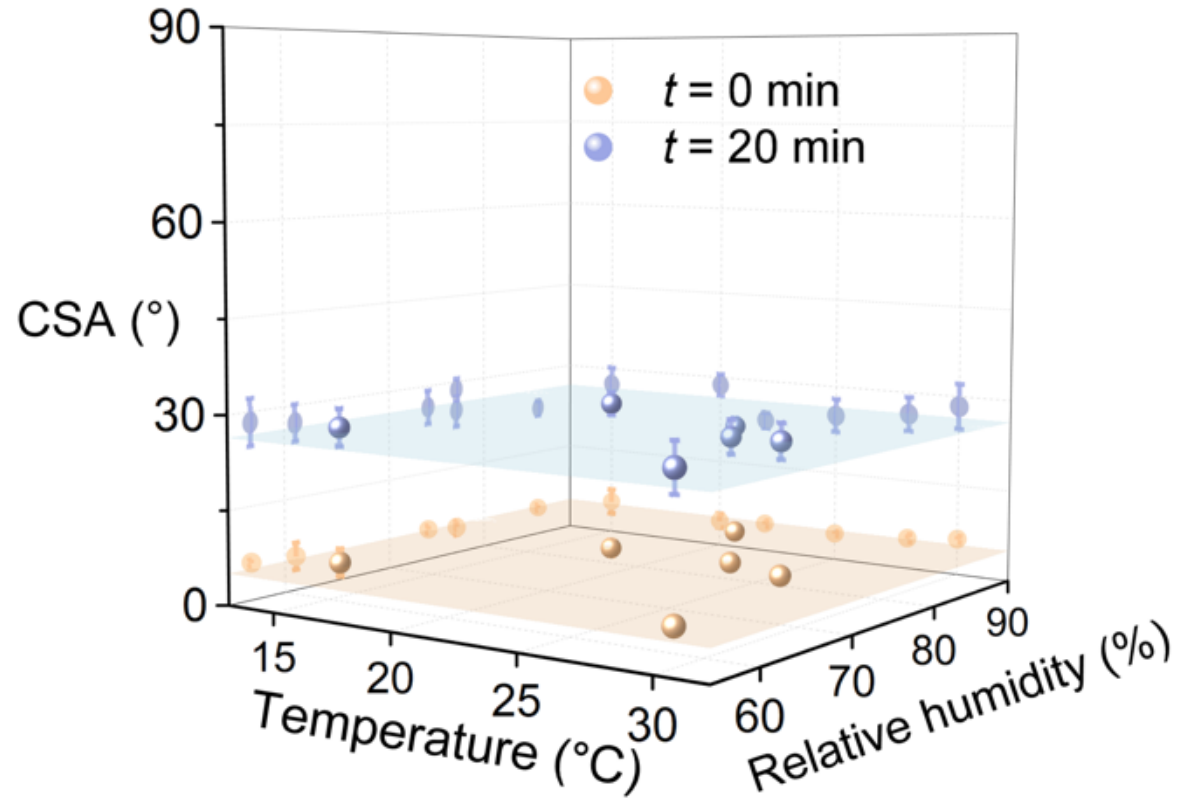

Fig. S11. The CSA of BSA droplets across varying test temperatures and relative humidities, demonstrating the method's stability for POCT.

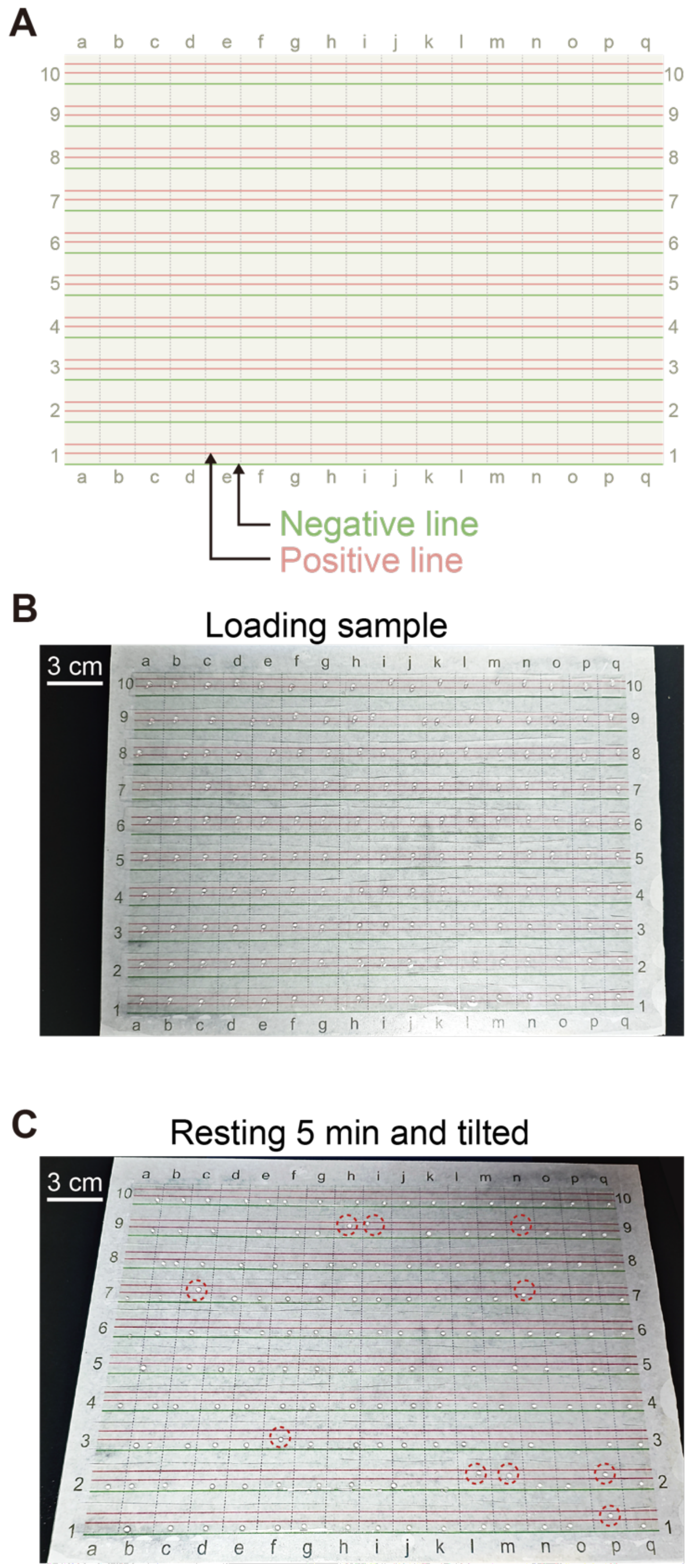

Fig. S12. Mass screening demonstration: (A) Schematic diagram of protein detection test table. (B-C) Images of assessment experiment: (B) Initial state of sample droplets after loading; (C) Droplets after 5 min static incubation followed by 20°-tilting. The red dashed circles indicate a fibrinogen droplet (1 g L^{-1}), while the remaining droplets are non-protein control.

Table S1. Capillary tube parameters

Length (mm)	Outer diameter (mm)	Spring constant (μN mm^{-1})	Test range (μN)
98	0.28	100.3	0-98

Table S2. The properties of proteins*

Protein	Molecular weight (kDa)	Molecule length (nm)	Chemical formula	Grand average of hydropathicity
BSA	69.3	8.0	$C_{3071}H_{4826}N_{816}O_{927}S_{40}$	-0.426
Lyz	16.2	4.3	$C_{705}H_{1116}N_{214}O_{204}S_{12}$	0.150
Fbg	341	44.7	$C_{14894}H_{23114}N_{4248}O_{4723}S_{120}$	-0.744

* The three-dimensional structure of the protein was obtained from the Protein Data Bank (PDB; https://www.rcsb.org/) using its corresponding ID. The maximum protein length can be obtained by measuring the distance between the two most distal atoms (or residues) using the measurement tool.

Table S3. The cost for the single protein test

Material	Price (USD)	Amount	Cost (USD)
PDMS	~0.14 g^{-1}	~0.03 g	~0.004
Silicone oil	~0.057 mL^{-1}	~0.075 mL	~0.004
Total			**~0.008**

Movie S1

The CSA comparison of different droplets.
